# Neonatal Exposure to Low Doses of Diazinon: Long-Term Effects on Neural Cell Development and Acetylcholine Systems

**DOI:** 10.1289/ehp.11005

**Published:** 2007-12-13

**Authors:** Theodore A. Slotkin, Bethany E. Bodwell, Edward D. Levin, Frederic J. Seidler

**Affiliations:** 1 Department of Pharmacology & Cancer Biology and; 2 Department of Psychiatry & Behavioral Sciences, Duke University Medical Center, Durham, North Carolina USA

**Keywords:** acetylcholine, brain development, diazinon, organophosphate insecticides

## Abstract

**Background:**

The developmental neurotoxicity of organophosphate pesticides involves mechanisms other than their shared property of cholinesterase inhibition.

**Objectives:**

We gave diazinon (DZN) to newborn rats on postnatal days 1–4, using doses (0.5 or 2 mg/kg) spanning the threshold for barely detectable cholinesterase inhibition.

**Methods:**

We then evaluated the lasting effects on indices of neural cell number and size, and on functional markers of acetylcholine (ACh) synapses (choline acetyltransferase, presynaptic high-affinity choline transporter, nicotinic cholinergic receptors) in a variety of brain regions.

**Results:**

DZN exposure produced a significant overall increase in cell-packing density in adolescence and adulthood, suggestive of neuronal loss and reactive gliosis; however, some regions (temporal/occipital cortex, striatum) showed evidence of net cell loss, reflecting a greater sensitivity to neurotoxic effects of DZN. Deficits were seen in ACh markers in cerebrocortical areas and the hippocampus, regions enriched in ACh projections. In contrast, there were no significant effects in the midbrain, the major locus for ACh cell bodies. The striatum showed a unique pattern, with robust initial elevations in the ACh markers that regressed in adulthood to normal or subnormal values.

**Conclusions:**

These results indicate that developmental exposures to apparently nontoxic doses of DZN compromise neural cell development and alter ACh synaptic function in adolescence and adulthood. The patterns seen here differ substantially from those seen in earlier work with chlorpyrifos, reinforcing the concept that the various organophosphates have fundamentally different effects on the developmental trajectories of specific neurotransmitter systems, unrelated to their shared action as cholinesterase inhibitors.

The developmental neurotoxicity of organophosphate pesticides occurs at doses below the threshold for overt symptoms of intoxication, or even lower than that required for cholinesterase inhibition, the standard biomarker for exposure and risk assessment (reviewed by Slotkin 1999, 2004, 2005). These agents alter the trajectory of neurodevelopment and formation of neural circuits through a family of mechanisms targeting cell replication and differentiation, axonogenesis and synaptogenesis, and functional development of neurotransmitter and neurotrophin systems, culminating in adverse effects on behavioral performance. Although the organophosphates damage a wide variety of neurotransmitter networks, those involving acetylcholine (ACh) nevertheless represent a major target, in part because some of these agents specifically redirect differentiation away from expression of the cholinergic phenotype (Jameson et al. 2006; Slotkin et al. 2001, 2007b) and also because developing cholinergic neurons and nerve terminals may be especially vulnerable to toxicant damage (Slotkin 1999, 2004, 2005). Accordingly, tests of cognitive performance, which depend heavily upon cholinergic function, readily reveal the adverse effects of developmental exposure to organophosphates (Chanda and Pope 1996; Jett et al. 2001; Levin et al. 2001). The same conclusions have been reached from behavioral tests of children living in environments with high organophosphate levels (Guillette et al. 1998; Landrigan 2001; Rauh et al. 2006).

The fact that organophosphate effects on brain development involve mechanisms other than their common feature of cholinesterase inhibition raises the likelihood that different members of this pesticide class could evoke dissimilar neurodevelopmental outcomes, so that some may pose greater neurotoxic risk than others at the same low-level exposures. This is a significant concern: Whereas there is a vast literature on the effects of chlorpyrifos, there is much less information available about the consequences of fetal or neonatal exposure to other organophosphates in widespread use, such as diazinon (DZN). Previous work both *in vitro* and *in vivo* suggests that there are similarities, but also substantial differences, in the initial effects of these two agents as well as in their ultimate neurobehavioral consequences (Jameson et al. 2007; Qiao et al. 2001, 2003, 2004; Slotkin et al. 2006a, 2007b, 2007c; Slotkin and Seidler 2007a; Timofeeva et al. 2008). In the present study, we focused on the effects of neonatal DZN exposure on indices of neural cell development and ACh systems in adolescence and adulthood, modeling our measures after earlier studies with chlorpyrifos (Campbell et al. 1997; Dam et al. 1999; Qiao et al. 2002, 2003, 2004; Rhodes et al. 2004; Slotkin et al. 2001, 2006a). We administered DZN during the immediate postnatal period [postnatal days (PNDs) 1–4], a stage at which we previously found high sensitivity of ACh systems to disruption by chlorpyrifos. Because of our interest in mechanisms other than cholinesterase inhibition, we evaluated two apparently nonsymptomatic DZN regimens (Slotkin et al. 2006a, 2006b; Slotkin and Seidler 2007a): 0.5 mg/kg/day, which produces no discernible cholinesterase inhibition, and 2 mg/kg/day, which elicits approximately 20% inhibition, equivalent to that obtained with 1 mg/kg/day of chlorpyrifos as used in our earlier work (Song et al. 1997). Because many of the developmental effects of chlorpyrifos are strongly sex-selective (Aldridge et al. 2004, 2005a; Dam et al. 2000; Levin et al. 2001, 2002; Slotkin 2005; Slotkin et al. 2001, 2002; Slotkin and Seidler 2007b), we evaluated both males and females for comparable effects of DZN.

To characterize the effects on neural cell development, we conducted measurements of DNA and cell protein fractions that characterize cell-packing density, cell number, and cell size. The DNA concentration (DNA per unit tissue weight), reflects the cell-packing density, whereas the DNA content (DNA per brain region) indicates the total number of cells (Bell et al. 1987; Slotkin et al. 1984; Winick and Noble 1965). The ratio of total protein/DNA rises as the size of the cell increases (Bell et al. 1987; Slotkin et al. 1984). The relative cell-membrane surface area, evaluated by the ratio of membrane protein/total protein changes in two ways. Replacement of neurons with smaller glial cells results in an increase in the surface/volume ratio and a corresponding rise in the membrane/total protein ratio. However, the development of neuritic projections also necessitates an increase in the contribution of membrane proteins relative to other cell proteins (Campbell et al. 1997; Qiao et al. 2002, 2003; Slotkin et al. 2007a, 2007b; Song et al. 1998).

For effects on ACh synaptic function, we assessed three markers: activity of choline acetyltransferase (ChAT), cell membrane binding of hemicholinium-3 (HC3) to the presynaptic high-affinity choline transporter, and the concentration of α4β2 nicotinic acetylcholine receptors (nAChRs). ChAT, the enzyme that synthesizes acetylcholine, is a constitutive component of cholinergic nerve terminals and thus provides a measure of the development of cholinergic projections (Dam et al. 1999; Happe and Murrin 1992; Monnet-Tschudi et al. 2000; Qiao et al. 2003; Richardson and Chambers 2005; Slotkin et al. 2001). In contrast, HC3 binding to the choline transporter is responsive to neuronal activity (Klemm and Kuhar 1979; Simon et al. 1976), so that measurement of both parameters enables the distinction between effects on the development of innervation from those on synaptic activity. These markers have been used previously to characterize effects of chlorpyrifos on ACh systems in adult rats (Liu and Pope 1996, 1998) and to evaluate the immediate and delayed effects of postnatal chlorpyrifos exposure (Dam et al. 1999; Rhodes et al. 2004; Richardson and Chambers 2005; Slotkin et al. 2001). Finally, the α4β2 nAChR is the most abundant nAChR subtype in the mammalian brain (Flores et al. 1992; Happe et al. 1994; Whiting and Lindstrom 1987, 1988); this receptor plays important roles in the ability of ACh systems to release other neurotransmitters involved in critical pathways such as those regulating reward, cognition, and mood (Buisson and Bertrand 2001, 2002; Dani and De Biasi 2001; Fenster et al. 1999; Quick and Lester 2002).

## Methods

### Animal treatments

All experiments were carried out humanely and with regard for alleviation of suffering, with protocols approved by the Institutional Animal Care and Use Committee and in accordance with all federal and state guidelines. Timed-pregnant Sprague-Dawley rats (Charles River, Raleigh, NC) were housed in breeding cages, with a 12-hr light–dark cycle and free access to food and water. On the day after birth, all pups were randomized and redistributed to the dams with a litter size of 10 (five males, five females) to maintain a standard nutritional status. Because of its poor water solubility, DZN (Chem Service, West Chester, PA) was dissolved in dimethylsulfoxide to provide consistent absorption (Slotkin et al. 2006a, 2006b; Slotkin and Seidler 2007a; Whitney et al. 1995) and was injected subcutaneously in a volume of 1 mL/kg once daily on PNDs 1–4; control animals received equivalent injections of the dimethylsulfoxide vehicle, which does not itself produce developmental neurotoxicity (Whitney et al. 1995). Doses of 0.5 and 2 mg/kg/day were chosen because they lie below the threshold for signs of systemic toxicity in developing rats, as evidenced by impaired viability or reduced weight gain (Slotkin et al. 2006a), and they straddle the threshold for barely detectable cholinesterase inhibition (Slotkin and Seidler 2007a; Slotkin et al. 2006b). These treatments thus resemble the nonsymptomatic exposures reported in pregnant women (De Peyster et al. 1993) and are pharmacodynamically comparable to expected fetal and childhood exposures after routine home application or in agricultural communities (Gurunathan et al. 1998; Ostrea et al. 2002). Randomization of pup litter assignments within treatment groups was repeated at intervals of several days up until weaning; in addition, dams were rotated among litters to distribute any maternal caretaking differences randomly across litters and treatment groups. Offspring were weaned on PND21.

On PNDs 30, 60, and 100, one male and one female were selected from each litter of origin and were decapitated. The cerebellum (including flocculi) was removed, and the midbrain/brainstem was separated from the fore-brain by a cut rostral to the thalamus. The striatum and hippocampus were then dissected from these larger divisions, and the midbrain and brainstem were divided from each other. The cerebral cortex was divided down the mid-line and then further sectioned into anterior and posterior regions (frontal/parietal cortex and temporal/occipital cortex, respectively). The experiments in the present study were performed on the frontal/parietal cortex and temporal/occipital cortex, the striatum, and the hippocampus, all of which contain the major ACh projections, and the midbrain, which contains the cell bodies projecting to the other regions. The brainstem was used solely for determinations of DNA and proteins, whereas the cerebellum was not evaluated, because it is sparse in ACh projections or cell bodies. Tissues were frozen with liquid nitrogen and stored at −45°C.

### Markers of neural cell number and size

Each tissue was thawed and homogenized (Polytron, Brinkmann Instruments, Westbury, NY) in ice-cold 10-mM sodium–potassium phosphate buffer (pH 7.4), and aliquots of the homogenate were withdrawn for measurement of DNA and total protein. DNA was assessed with a fluorescent dye-binding method (Trauth et al. 2000). Aliquots were diluted in 50 mM sodium phosphate, 2 M NaCl, and 2 mM EDTA (pH 7.4) and sonicated briefly (Virsonic Cell Disrupter, Virtis, Gardiner, NY). Hoechst 33258 (Sigma Chemical Co., St. Louis, MO) was added to a final concentration of 1 μg/mL. Samples were then read in a spectrofluorometer using an excitation wavelength of 356 nm and an emission wavelength of 458 nm, and were quantitated using standards of purified DNA. The total concentration of tissue proteins was assayed spectrophotometrically by dye binding (Smith et al. 1985); in addition, we assessed the concentration of membrane proteins from the membrane preparations used for radioligand binding, as described below.

### ACh markers

Aliquots of the same homogenate used for DNA determinations were assayed in duplicate for ChAT using established procedures (Qiao et al. 2003, 2004). Each tube contained final concentrations of 60 mM sodium phosphate (pH 7.9), 200 mM NaCl, 20 mM choline chloride, 17 mM MgCl_2_, 1 mM EDTA, 0.2% Triton X-100, 0.12 mM physostigmine, 0.6 mg/mL bovine serum albumin, and 50 μM [^14^C]acetyl-coenzyme A. Blanks contained homogenization buffer instead of the tissue homogenate. Samples were preincubated for 15 min on ice and transferred to a 37°C water bath for 30 min; the reaction was terminated by placing the samples on ice. Labeled acetylcholine was then extracted and counted, and the activity was determined relative to tissue protein (Smith et al. 1985). Preliminary determinations established that enzyme activity was linear with time and tissue concentration under these conditions.

For measurements of HC3 binding, an aliquot of the same tissue homogenate was sedimented at 40,000 × *g* for 15 min and the supernatant solution was discarded. The membrane pellet was resuspended (Polytron) in the original volume of buffer and resedimented, and the resultant pellet was resuspended using a smooth glass homogenizer fitted with a Teflon pestle, in 10 mM sodium–potassium phosphate buffer (pH 7.4) containing 150 mM NaCl. An aliquot was withdrawn for the determination of membrane protein (Smith et al. 1985), and radioligand binding was evaluated with 2 nM [^3^H]HC3 (Qiao et al. 2003, 2004), with incubation for 20 min at room temperature, followed by rapid vacuum filtration onto glass fiber filters (presoaked for 30 min with 0.15% polyethyleneimine in buffer). The nonspecific component was defined as radioligand binding in the presence of an excess concentration of unlabeled HC3 (10 μM), and binding values were expressed relative to membrane protein. For nAChR binding, each assay contained a final concentration of 1 nM [^3^H]cytisine in a total volume of 250 μL of a buffer consisting of 120 mM NaCl, 5 mM KCl, 2.5 mM CaCl_2_, 1 mM MgCl_2_, and 50 mM Tris (pH 7.4). Incubations lasted 75 min at 4°C, with or without 10 μM nicotine to displace specific binding.

### Data analysis

Data were compiled as means and SEs. Because we evaluated multiple neurochemical variables that were all related to cell number and size, or to ACh synapses, the initial comparisons were conducted by a global analysis of variance (ANOVA; data were log-transformed because of heterogeneous variance among ages, regions, and measures) incorporating all the variables and measurements for each of the two classes so as to avoid an increased probability of type 1 errors that might otherwise result from multiple tests of the same data set: for cell development parameters, DNA per gram of tissue, DNA per region, total protein per DNA ratio, membrane protein per total protein ratio; for ACh synapses, ChAT activity, HC3 binding, and nAChR binding. Where we identified interactions of treatment with the other variables, data were then subdivided for lower-order ANOVAs to evaluate treatments that differed from the corresponding control; where permitted by the interaction terms, individual groups that differed from the corresponding control values in a given region at a given age were identified with Fisher’s protected least-significant difference test. Significance was assumed at the level of *p* < 0.05 for main effects; however, for interactions at *p* < 0.1, we also examined whether lower-order main effects were detectable after subdivision of the interactive variables (Snedecor and Cochran 1967). For convenience, some of the results are presented as the percent change from control values, but statistical comparisons were conducted only on the original data. The corresponding control values are shown in Tables 1 and 2, so the reader can readily derive the original values.

## Results

### Neural cell development markers

Neonatal DZN treatment did not elicit any deficits in body or brain region weights (data not shown). Nevertheless, we found significant effects on the markers for neural cell number and size in adolescence and adulthood. Global repeated-measures ANOVA incorporating all the cell-development variables (treatment, age, region, sex, four measures) indicated a main treatment effect for DZN (*p* < 0.03), reflecting a significant overall difference for the low-dose DZN group compared with either controls (*p* < 0.03) or the high-dose DZN group (*p* < 0.003). In addition, the treatment effect showed interactions with region, age, and measure: *p* < 0.02 for treatment × region; *p* < 0.08 for treatment × age; *p* < 0.02 for treatment × measure; and *p* < 0.05 for treatment × region × measure. Accordingly, data were subdivided into individual measures (DNA concentration, DNA content, total protein/DNA, membrane protein/total protein) and reexamined for treatment effects and interactions with the remaining variables. Because there were no interactions of treatment × sex or treatment × sex × other variables, treatment effects are shown combined for males and females; however, the sex factor was retained in performing the lower-order statistical analyses.

**Figure 1:**
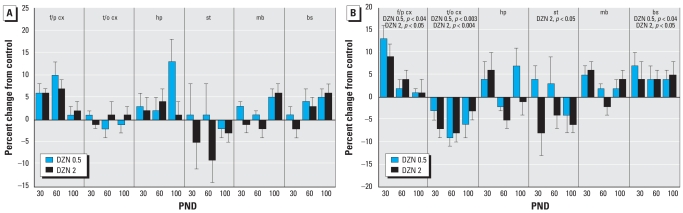
Effects of neonatal DZN exposure on indices of cell-packing density and total cell number. (*A*) DNA concentration (DNA per unit tissue weight). (*B*) DNA content (DNA per brain region). Abbreviations: bs, brain stem; f/p cx, frontal/parietal cortex; hp, hippocampus; mb, midbrain; st, striatum; t/o cx, temporal/occipital cortex. Data represent means ± SEs obtained from six animals of each sex in each treatment group at each age, presented as the percentage change from control values shown in Table 1. Values for males and females were combined because of the absence of interactions of treatment × sex or treatment × sex × other variables. Multivariate ANOVA for all factors (all treatments, both sexes, all ages, all regions) were performed. In (*A*), there was an overall main treatment effect (*p* < 0.02), with significant differences from the control restricted to the DZN 0.5 group (*p* < 0.009). Further subdivisions of the data were not examined because of the absence of interactions of treatment with the other variables. In (*B*), treatment × region is significant at *p* < 0.004, and lower-order ANOVAs for each region appear within the panel; subdivision into separate ages was not carried out because of the absence of an interaction of treatment × age.

For DNA concentration, we observed a small but highly statistically significant overall increase at the lower DZN dose (Figure 1A). The effect was less notable at the higher dose, which did not show statistically significant differences from the control group; in fact, the increase seen at the low dose was itself distinguishable from the lack of effect in the high-dose group (*p* < 0.04). Although increases were also seen for DNA content, the marker for total neural cell numbers, there were significant regional distinctions (Figure 1B). Values were increased with either 0.5-mg/kg or 2-mg/kg DZN in the frontal/parietal cortex and the brainstem, whereas there were decreases at either dose in the temporal/occipital cortex and at the higher dose in the striatum.

**Figure 2:**
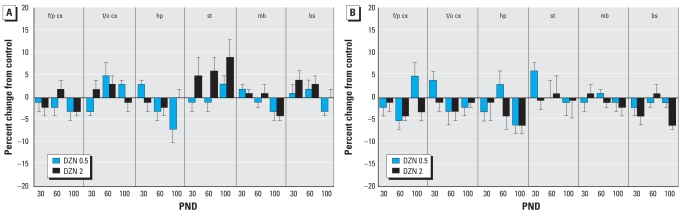
Effects of neonatal DZN exposure on indices of cell size. (*A*) Total protein/DNA ratio. (*B*) Membrane protein/total protein ratio. Abbreviations: bs, brain stem; f/p cx, frontal/parietal cortex; hp, hippocampus; mb, midbrain; st, striatum; t/o cx, temporal/occipital cortex. Data represent means ± SEs obtained from six animals of each sex in each treatment group at each age, presented as the percentage change from control values shown in Table 1. Values for males and females were combined because of the absence of interactions of treatment × sex or treatment × sex × other variables. Multivariate ANOVA for all factors (all treatments, both sexes, all ages, all regions) were performed. In (*A*), there was an overall main treatment effect (*p* < 0.05), as well as an interaction of treatment × region (*p* < 0.07). The main effect was significant for the DZN 2 group (*p* < 0.05) and lower-order ANOVAs for each region identified a significant difference only in the striatum (*p* < 0.03). Subdivision into separate ages was not carried out because of the absence of an interaction of treatment × age. In (*B*), there was an overall main treatment effect (*p* < 0.05), with significant differences from the control restricted to the DZN 2 group (*p* < 0.03). Further subdivisions of the data were not examined because of the absence of interactions of treatment with the other variables.

The effects on protein-related markers were far less notable at the low dose of DZN, as neither the total protein/DNA ratio (Figure 2A) nor the membrane protein/total protein ratio (Figure 2B) showed significant differences. Raising the DZN dose to 2 mg/kg revealed a significant (*p* < 0.05) overall increase in the total protein/DNA ratio that was particularly prominent in the striatum (Figure 2A); there was also a significant (*p* < 0.03) overall decrement in the membrane protein/total protein ratio (Figure 2B).

### ACh markers

Global ANOVA for the ACh markers (treatment, age, region, sex, three measures) identified interactions of DZN treatment with the other variables, in this case, necessitating separation of effects on males and females: *p* < 0.04 for treatment × age × sex; *p* < 0.07 for treatment × age × region; *p* < 0.03 for treatment × region × measure; *p* < 0.002 for treatment × age × region × measure; *p* < 0.02 for treatment × sex × region × measure; and *p* < 0.08 for treatment × age × sex × region × measure. Thus, for these variables, we performed lower-order analyses on each measure separately for each region and sex.

**Figure 3:**
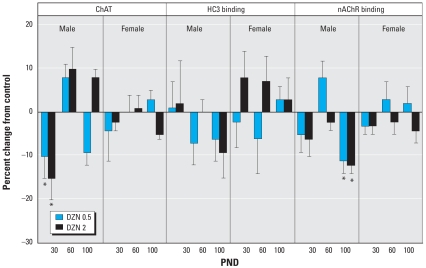
Effects of neonatal DZN exposure on cholinergic markers in the frontal/parietal cortex. Data represent means ± SEs obtained from six animals of each sex in each treatment group at each age, presented as the percentage change from control values shown in Table 2. Multivariate ANOVA for all factors (all treatments, both sexes, all ages, all measures): *p* < 0.02 for treatment × age × sex × measure. Because treatment interacted with all the other variables, separate tests were conducted for each treatment and measure at each age, separated by sex. *Significantly different from control values.

**Figure 4:**
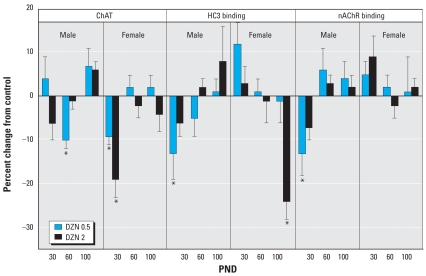
Effects of neonatal DZN exposure on cholinergic markers in the temporal/occipital cortex. Data represent means ± SEs obtained from six animals of each sex in each treatment group at each age, presented as the percentage change from control values shown in Table 2. Multivariate ANOVA for all factors (all treatments, both sexes, all ages, all measures): *p* < 0.04 for treatment × sex × measure, and *p* < 0.03 for treatment × age × sex × measure. Because treatment interacted with all the other variables, separate tests were conducted for each treatment and measure at each age, separated by sex. *Significantly different from control values.

**Figure 5:**
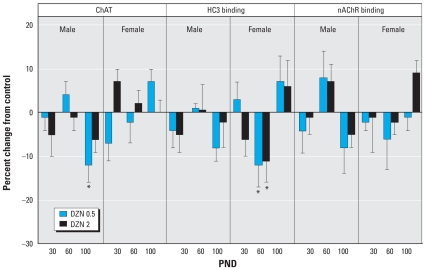
Effects of neonatal DZN exposure on cholinergic markers in the hippocampus. Data represent means ± SEs obtained from six animals of each sex in each treatment group at each age, presented as the percentage change from control values shown in Table 2. Multivariate ANOVA for all factors (all treatments, both sexes, all ages, all measures): *p* < 0.03 for treatment × age × sex, and *p* < 0.07 for treatment × age × sex × measure. Because treatment interacted with all the other variables, separate tests were conducted for each treatment and measure at each age, separated by sex. *Significantly different from control values.

In the frontal/parietal cortex, we found substantial decreases in ChAT in adolescence (PND30) in males exposed to DZN but no apparent changes in HC3 binding, the index of presynaptic neural activity (Figure 3). At PND100, the only significant effect was a decrease in nAChR binding, again restricted to males. More robust effects were seen in the temporal/occipital cortex (Figure 4). In males, there were deficits in adolescence or early adulthood for ChAT (PND60), HC3 binding (PND30), and nAChR binding (PND30), again displaying more preponderant effects at the low-DZN dose rather than the high dose. In females, there were robust decreases in ChAT and nAChR binding at PND30 and a large deficit in HC3 binding at PND100. Effects on the hippocampus differed from those in the cerebrocortical regions in that there was little or no effect apparent early on (PND30), but deficits emerged in adulthood (Figure 5). Males showed deficiencies in ChAT on PND100 and females showed a reduction in HC3 binding at PND60.

**Figure 6:**
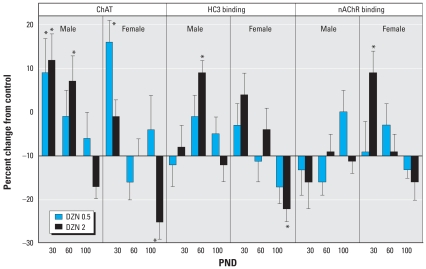
Effects of neonatal DZN exposure on cholinergic markers in the striatum. Data represent means ± SEs obtained from six animals of each sex in each treatment group at each age, presented as the percentage change from control values shown in Table 2. Multivariate ANOVA for all factors (all treatments, both sexes, all ages, all measures): *p* < 0.003 for treatment × age, *p* < 0.02 for treatment × measure, and *p* < 0.004 for treatment × age × measure. Because treatment interacted with all the other variables, separate tests were conducted for each treatment and measure at each age, separated by sex. *Significantly different from control values.

**Figure 7:**
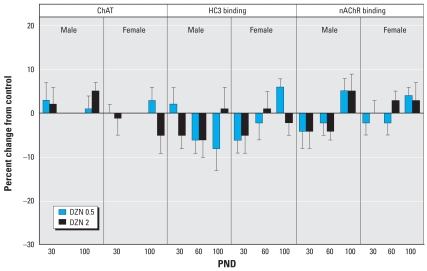
Effects of neonatal DZN exposure on cholinergic markers in the midbrain. Data represent means ± SEs obtained from six animals of each sex in each treatment group at each age, presented as the percentage change from control values shown in Table 2. Multivariate ANOVAs for all factors (all treatments, both sexes, all ages) were conducted separately for each of the measures because of the missing values for ChAT on PND60; none of these were significant. Accordingly, no lower-order tests on subdivisions of the data were carried out.

The striatum showed unique and robust effects of DZN (Figure 6). On PND30, we observed marked increases in ChAT activity in both males and females; although the effect persisted in males through PND60, both sexes showed regression by PND100. At that point, ChAT was reduced in females at the high-DZN dose, an effect that was paralleled by a decrease in HC3 binding. Males displayed a transient increase in hippocampal HC3 binding (PND60) that was no longer evident by PND100. For nAChR binding, the only effect was an increase at PND30 in females exposed to the high-DZN dose. We observed no significant effects on ACh markers in the midbrain, the location of many of the ACh cell bodies that project to the other regions (Figure 7).

## Discussion

Behavioral evaluations of adolescent and adult rats exposed as neonates to DZN doses below or just above the threshold for detectable cholinesterase inhibition indicate lasting deficits in cognitive performance and alterations in emotional responsiveness (Roegge et al. 2008; Timofeeva et al. 2008). Results of the present study show that these changes are associated with corresponding effects on indices of neural cell development and ACh synaptic function. For the cell development markers, the low dose of DZN, which does not cause any discernible cholinesterase inhibition (Slotkin et al. 2006b), evoked a small but consistent increase in the DNA concentration in various brain regions, indicative of an increase in cell-packing density (i.e., more cells per unit volume). Typically, this reflects a loss of neurons and their replacement by smaller glial cells (O’Callaghan 1988); in keeping with this interpretation, the total number of cells (DNA content) showed a significant increase in two of the regions (frontal/parietal cortex, brainstem), as would be expected from reactive gliosis. In contrast, one region (temporal/occipital cortex) showed a decrease in DNA content, indicative of even greater net toxicity that compromises the total number of cells. In turn, this suggests a biphasic response, with neuronal loss and glial “scarring” in the less sensitive regions, and loss of both neurons and glia in the more sensitive regions. Indeed, DZN is inherently more toxic toward glial cells than neurons (Qiao et al. 2001); however, glia, unlike neurons, can be replaced, so the net effect reflects the balance between damage and repair, which are likely to vary among brain regions simply because they differ in their maturational state at the time of exposure (Rodier 1988). This conclusion is strongly supported by the finding that increasing the dose of DZN to 2 mg/kg reduced the effect on cell-packing density to nonsignificance and enhanced the net cell loss as exemplified by DNA content, extending the deficits to include the striatum. The biphasic effect is thus suggestive of incrementally greater gliotoxicity at the higher dose, in agreement with prior measurements of the expression of gliotypic genes in the immediate postexposure period (Slotkin and Seidler 2007a). Obviously, though, quantitative morphologic examinations will be required to provide complete proof of these conclusions, as has been done for chlorpyrifos (Slotkin 1999, 2004, 2005): Homogenization of brain regions containing diverse neuronal groupings means that even drastic effects on a specific population of neurons may go unnoticed because of dilution with unaffected areas (Qiao et al. 2002, 2003). Despite this limitation, we found statistically significant alterations in biomarkers of cell number and cell-packing density, indicating that much larger changes are likely to be present in more restricted neuroanatomical locations.

In contrast to the effects on indices of cell-packing density and cell number, the markers related to cell growth and size were less notably affected by DZN exposure and were limited to effects at the higher dose. There was a small but significant overall increase in total protein/DNA, achieving statistical significance in one region (striatum), as well as a small overall decrease in membrane/total protein. The main point is that DZN exposure targets cell numbers more than it targets cell growth. Nevertheless, the protein measures reinforce some of the conclusions from the DNA measurements; the increase in total protein/DNA in the striatum is the opposite of what would be expected from an elevation in the number of glial cells, because the smaller glia would have lower protein per cell. Similarly, the small decrease in membrane/total protein is suggestive of a negative impact on generation of neuritic projections, effects that have already been clearly demonstrated for DZN *in vitro* (Axelrad et al. 2002, 2003). Furthermore, in earlier work, we found that neonatal DZN exposure *in vivo* immediately shifts the expression of genes required for the generation of neurites (Slotkin and Seidler 2007a) and produces a drop in membrane/total protein prior to the stage at which glia are being generated (Slotkin et al. 2006a). It is thus highly likely that DZN, like chlorpyrifos (Axelrad et al. 2003; Das and Barone 1999; Howard et al. 2005), compromises the development of neuronal projections. Accordingly, there is no reason to suspect that adverse effects will be limited to the ACh systems studied here, even though, as described below, there is profound disruption of ACh functional indices. Indeed, we have already shown that there are both immediate and lasting deficits in serotonergic synaptic function after apparently subtoxic neonatal DZN exposures (Slotkin et al. 2006b, in press; Slotkin and Seidler 2007a).

In the two cerebrocortical regions and the hippocampus, DZN elicited profound deficits in all three ACh synaptic markers in either adolescence or adulthood, with differing profiles according to region. These findings all point toward deficiencies in ACh innervation, ACh synaptic activity, or ACh receptor expression, producing a net outcome of impairment of ACh synaptic function. In the frontal/parietal cortex, the deficits were seen in males, whereas both sexes showed adverse effects in the temporal/occipital cortex. In the hippocampus, the deficits emerged in adulthood after apparent normality in adolescence; this reinforces the idea that developmental neurotoxicant exposures can act in two distinct ways, producing immediate damage or changing the trajectory of neurodevelopment so that alterations appear after a substantial delay. The hippocampal and cortical ACh impairments are particularly important in light of the cognitive and emotional dysfunction seen with this DZN exposure model (Roegge et al. 2008; Timofeeva et al. 2008). The nonmonotonic dose–effect relationship for effect on ACh systems precisely mirrors that seen for cognitive impairment (Timofeeva et al. 2008). In that study, we found that the animals given the low dose of DZN were sensitized to the amnestic effects of scopolamine, a muscarinic ACh antagonist, results entirely in keeping with compromised ACh function as seen here.

In contrast to the other regions containing major ACh projections, the striatum showed an entirely different pattern of effects, with initial increases in the ACh markers, followed by a regression to normal or subnormal values at later stages. The striatum differs in several substantial ways; for example, striatal ACh neurons are short-axon interstitial cells rather than the long-projection neurons found in the other regions. Furthermore, its high concentration of dopaminergic projections conveys a consequently greater susceptibility to oxidative stress, including that elicited by pesticide exposures (Hirsch 1994; Junn and Mouradian 2001; Karen et al. 2001; Kitazawa et al. 2001; Olanow and Arendash 1994). In comparative studies of gene expression patterns and markers of the cell damage/repair response, we found that DZN has a greater propensity to elicit these types of effects than does chlorpyrifos (Jameson et al. 2007; Slotkin and Seidler 2007a), so the striatum may represent a more specific regional target for DZN. Further, neonatal DZN exposure interferes with the expression of specific neurotrophic factors that govern the development of striatal dopamine systems (Slotkin et al. 2007c). Our results therefore suggest that DZN evokes a significant loss of dopaminergic projections and/or dopaminergic function, and a consequent invasion of dopaminergic terminal zones with ACh. Indeed, we have seen just this type of neurotransmitter replacement for the loss of hippocampal ACh function after developmental exposure to chlorpyrifos, where serotonergic projections instead take over the innervation and function of nominally cholinergic terminal zones (Aldridge et al. 2005a, 2005b; Icenogle et al. 2004; Levin et al. 2001, 2002). It would clearly be worthwhile to expand the scope of investigation to include dopamine systems; developmental loss of dopamine projections as a result of environmental toxicant exposure are suspected to underlie the later emergence of Parkinson disease (Cory-Slechta et al. 2005; Kamel and Hoppin 2004; Landrigan et al. 2005), and our findings of a rapid decline in striatal ACh markers in adulthood points to a progressive loss of other aspects of synaptic function in this region.

Because we observed adverse effects of DZN at exposures below the threshold for any detectable cholinesterase inhibition, it is likely that the underlying cellular mechanisms involve other actions on specific processes involved in neural cell replication and differentiation (Barone et al. 2000; Betancourt et al. 2006; Betancourt and Carr 2004; Casida and Quistad 2004; Gupta 2004; Pope 1999; Qiao et al. 2002, 2003; Slotkin 1999, 2004, 2005; Slotkin et al. 2007c; Yanai et al. 2002). If this is true, then there is no reason to expect that the effects of DZN will be the same as those of other organophosphates, such as chlorpyrifos, even when selected doses are dynamically equivalent, that is, that occupy the same part of the dose–response curve just below and at the threshold for cholinesterase inhibition. Indeed, our results indicate just that. Whereas DZN increased the cell-packing density, chlorpyrifos does the opposite (Qiao et al. 2003), representing its greater propensity for gliotoxicity (Garcia et al. 2001; Qiao et al. 2001). Although both DZN and chlorpyrifos (Qiao et al. 2003; Slotkin et al. 2001) reduce ChAT activity in cerebrocortical areas and hippocampus in adolescence and adulthood, they differ in their regional targeting and sex selectivity. Strikingly, whereas we found that DZN elevated many of the striatal ACh markers in either adolescence or young adulthood, chlorpyrifos has the opposite effect (Qiao et al. 2003; Slotkin et al. 2001). As one point of convergence, both DZN and chlorpyrifos largely spare the midbrain, which contains higher concentrations of ACh cell bodies as distinct from terminals. Nevertheless, given the large disparities in their effects in the brain regions containing the major ACh projections, these results do provide a mechanistic underpinning for the notable differences in behavioral outcomes after otherwise equivalent neonatal exposures to these two organophosphates (Aldridge et al. 2005a; Levin et al. 2001, 2002; Roegge et al. 2008; Timofeeva et al. 2008).

In conclusion, early postnatal exposure to DZN, at doses insufficient to cause any discernible signs of exposure and below the threshold for inhibition of cholinesterase, nevertheless elicits lasting changes in indices of neural cell development and ACh synaptic function. Together with our recent behavioral studies showing impairment of cognitive function and altered emotional responses (Roegge et al. 2008; Timofeeva et al. 2008), these results point to the inadequacy of cholinesterase measurements alone as a biomarker for defining the safe exposure limits for developmental neurotoxicity of organophosphates. Further, the nonmonotonic dose–effect relationships seen for both DZN and chlorpyrifos in terms of ACh systems and corresponding behavioral outcomes highlight the importance of examining dose ranges well below those required for cholinesterase inhibition. Given the variety of mechanisms of action of these compounds, toxic effects may be unmasked at lower doses where potentially counteracting secondary effects are no longer taking place. Finally, the dichotomous outcomes of exposure to DZN compared with chlorpyrifos reinforce the conclusion that although organophosphates, as a class, target neural cell development and ACh systems, each organophosphate is likely to elicit a unique pattern of damage/repair and altered synaptic function.

## Figures and Tables

**Table 1:** Control values for cell development markers.

		PND30		PND60		PND100	
Measure	Region	Male	Female	Male	Female	Male	Female
DNA concentration (μg/g tissue)	f/p cx	1,053 ± 24	1,088 ± 32	937 ± 26	903 ± 33	829 ± 25	868 ± 17
	t/o cx	775 ± 17	844 ± 21*	749 ± 17	705 ± 22	813 ± 22	828 ± 16
	hp	752 ± 12	760 ± 32	681 ± 31	680 ± 37	674 ± 25	794 ± 30*
	st	816 ± 47	855 ± 76	928 ± 96	975 ± 90	864 ± 42	740 ± 36
	mb	851 ± 10	898 ± 11*	759 ± 16	795 ± 14	863 ± 17	893 ± 23
	bs	797 ± 15	802 ± 28	756 ± 12	786 ± 13	621 ± 9	670 ± 17*
DNA content (μg/region)	f/p cx	542 ± 28	562 ± 18	608 ± 24	576 ± 42	580 ± 8	538 ± 18
	t/o cx	264 ± 7	275 ± 4	267 ± 6	271 ± 7	253 ± 12	255 ± 7
	hp	75 ± 4	80 ± 3	79 ± 3	81 ± 3	85 ± 4	86 ± 5
	st	80 ± 2	77 ± 3	113 ± 8	109 ± 5	116 ± 4	93 ± 6*
	mb	222 ± 8	223 ± 2	229 ± 8	231 ± 7	308 ± 12	299 ± 10
	bs	113 ± 4	112 ± 3	149 ± 2	149 ± 7	152 ± 5	144 ± 3
Total protein/DNA (μg/μg)	f/p cx	81 ± 2	80 ± 2	97 ± 2	97 ± 2	92 ± 3	93 ± 3
	t/o cx	88 ± 1	86 ± 1	106 ± 7	119 ± 6	102 ± 3	98 ± 3
	hp	83 ± 2	78 ± 2	110 ± 3	113 ± 6	117 ± 4	113 ± 2
	st	87 ± 3	102 ± 3*	89 ± 4	86 ± 4	85 ± 3	94 ± 4
	mb	89 ± 2	92 ± 1	109 ± 3	104 ± 3	97 ± 4	95 ± 2
	bs	96 ± 2	97 ± 3	110 ± 2	106 ± 2	136 ± 3	134 ± 2
Membrane protein/total protein (%)	f/p cx	38 ± 1	37 ± 1	53 ± 1	54 ± 1	53 ± 1	54 ± 1
	t/o cx	45 ± 2	44 ± 1	39 ± 1	39 ± 2	46 ± 1	46 ± 2
	hp	49 ± 1	49 ± 1	54 ± 2	55 ± 1	50 ± 2	46 ± 2
	st	48 ± 1	48 ± 3	46 ± 3	46 ± 2	57 ± 2	59 ± 2
	mb	50 ± 1	48 ± 1	46 ± 1	48 ± 1	56 ± 2	55 ± 1
	bs	48 ± 2	47 ± 1	46 ± 2	47 ± 1	46 ± 1	44 ± 2

Abbreviations: bs, brain stem; f/p cx, frontal/parietal cortex; hp, hippocampus; mb, midbrain; st, striatum; t/o cx, temporal/occipital cortex. Data are means ± SEs obtained from six animals of each sex at each age.

* Females significantly different from males.

**Table 2:** Control values for cholinergic markers.

		PND30		PND60		PND100	
Measure	Region	Male	Female	Male	Female	Male	Female
ChAT activity (pmol/min per mg protein)	f/p cx	763 ± 36	728 ± 42	706 ± 26	740 ± 29	785 ± 21	833 ± 18
	t/o cx	454 ± 29	458 ± 18	624 ± 17	595 ± 22	560 ± 18	592 ± 14
	hp	643 ± 28	611 ± 33	689 ± 2	685 ± 9	769 ± 14	774 ± 22
	st	573 ± 24	539 ± 39	864 ± 57	973 ± 37	1,318 ± 97	1,356 ± 63
	mb	775 ± 13	770 ± 24	—a	—a	620 ± 21	681 ± 5*
HC3 binding (fmol/mg protein)	f/p cx	15.9 ± 0.7	16.7 ± 1.4	14.1 ± 0.4	14.3 ± 0.5	14.1 ± 0.6	13.6 ± 0.8
	t/o cx	15.4 ± 0.8	15.5 ± 0.5	12.8 ± 0.8	12.2 ± 0.7	13.2 ± 0.4	16.5 ± 1.0*
	hp	26.6 ± 2.0	26.2 ± 1.0	19.3 ± 0.4	21.9 ± 0.8*	18.3 ± 0.7	17.8 ± 0.9
	st	71 ± 4	60 ± 2*	67 ± 3	74 ± 4	72 ± 2	76 ± 3
	mb	15.2 ± 0.7	15.4 ± 1.1	12.4 ± 0.3	12.9 ± 0.4	12.6 ± 0.4	12.3 ± 0.6
nAChR binding (fmol/mg protein)	f/p cx	66 ± 3	64 ± 2	62 ± 1	59 ± 2	53 ± 2	48 ± 2
	t/o cx	87 ± 4	78 ± 1	70 ± 2	70 ± 3	63 ± 2	66 ± 2
	hp	46 ± 3	45 ± 2	38 ± 2	40 ± 2	32 ± 1	29 ± 2
	st	88 ± 4	72 ± 3*	72 ± 1	68 ± 3	61 ± 1	60 ± 2
	mb	102 ± 3	96 ± 3	74 ± 3	71 ± 1	67 ± 2	67 ± 2

Abbreviations: f/p cx, frontal/parietal cortex; hp, hippocampus; mb, midbrain; st, striatum; t/o cx, temporal/occipital cortex. Data are means ± SEs obtained from six animals of each sex at each age.

a ChAT is missing because of a technical error for the samples on that day.

* Females significantly different from males.
